# Processing Mixed Mesopelagic Biomass from the North-East Atlantic into Aquafeed Resources; Implication for Food Safety

**DOI:** 10.3390/foods10061265

**Published:** 2021-06-02

**Authors:** Marc H. G. Berntssen, Lars Thoresen, Sissel Albrektsen, Eduardo Grimaldo, Leif Grimsmo, Ragnhild Dragøy Whitaker, Veronika Sele, Martin Wiech

**Affiliations:** 1 Institute of Marine Research, P.O. Box 1870 Nordnes, 5817 Bergen, Norway; Veronika.Sele@hi.no (V.S.); Martin.Wiech@hi.no (M.W.); 2 NOFIMA, Kjerreidvika 16, 5141 Fyllingsdalen, Norway; lars.thoresen@nofima.no (L.T.); sissel.albrektsen@nofima.no (S.A.); 3 SINTEF Ocean, Brattørkaia 17C, 7010 Trondheim, Norway; eduardo.grimaldo@sintef.no (E.G.); leif.grimsmo@sintef.no (L.G.); 4 NOFIMA, P.O. Box 6122 Langnes, 9291 Tromsø, Norway; Ragnhild.Whitaker@Nofima.no

**Keywords:** mesopelagic, contaminants, trace elements, arsenic, fluoride, organic pollutants, dioxins, PCB, farmed seafood, feed safety, food safety

## Abstract

Aquaculture produces most of the world’s seafood and is a valuable food source for an increasing global population. Low trophic mesopelagic biomasses have the potential to sustainably supplement aquafeed demands for increased seafood production. The present study is a theoretical whole-chain feed and food safety assessment on ingredients from mesopelagic biomass and the resulting farmed fish fed these ingredients, based on analysis of processed mesopelagic biomass. Earlier theoretical estimations have indicated that several undesirable compounds (e.g., dioxins and metals and fluoride) would exceed the legal maximum levels for feed and food safety. Our measurements on processed mesopelagic biomasses show that only fluoride exceeds legal feed safety limits. Due to high levels of fluoride in crustaceans, their catch proportion will dictate the fluoride level in the whole biomass and can be highly variable. Processing factors are established that can be used to estimate the levels of undesirables in mesopelagic aquafeed ingredients from highly variable species biomass catches. Levels of most the studied undesirables (dioxins, PCBs, organochlorine pesticides, brominated flame retardant, metals, metalloids) were generally low compared to aquafeed ingredients based on pelagic fish. Using a feed-to-fillet aquaculture transfer model, the use of mesopelagic processed aquafeed ingredients was estimated to reduce the level of dioxins and PCBs by ~30% in farmed seafood such as Atlantic salmon.

## 1. Introduction

Seafood from aquaculture is a valuable resource to meet the nutritional needs for a growing global population [1,2,3]. Traditionally, aquaculture feed has relied on fish oil and meal from wild caught pelagic fish (e.g., blue whiting (*Micromesistius poutassou*)) [4]. However, limited access to pelagic fish oil and fish meal has led to the search for alternative feed ingredients [2,5,6,7]. Unexploited marine resources, preferentially from lower trophic levels such as organisms from the mesopelagic zone [8], could supplement currently used feed ingredients [9,10]. However, the use of low trophic marine biomasses for aquafeed would depend on a sustainable harvest, and currently, little is known on the mesopelagic stocks. Commercial harvesting of this biomass would require assessment in order to set quotas that will not harm the marine ecosystem or the global CO_2_ budget and will thereby not facilitate climate change [11,12].

Processed agricultural plant products (soybean, wheat, and rapeseed) have supplanted a considerable fraction of pelagic fish oil and meal in aquafeeds [13]. However, the use of plant ingredients has introduced new risks to aquaculture (i.e., antinutrients, pesticides, and mycotoxins) [2] and the sustainability of continued agricultural areal expansion to produce plant protein and oils has been questioned [14]. Furthermore, the increased use of plant products has reduced the content of marine nutrients in farmed seafood such as very long chain n-3 poly unsaturated fatty acids and vitamin D and A, which are associated with health-benefits [15,16,17,18]. On the other hand, the use of plant products has also reduced the level of potential harmful environmental contaminants that follow marine aquafeed ingredients such as persistent organic pollutants (POPs) including dioxins (PCDDs), furans (PCDFs), dioxin-like polychlorinated biphenyls (DL-PCBs), organochlorine pesticides (OCPs) and polybrominated diphenyl ethers (PBDE), as well as and metals and metalloids such as arsenic (As), cadmium (Cd), mercury (Hg), and lead (Pb) [2,19,20]. The suggested use of mesopelagic marine resources in the now dominantly plant-based aquafeeds can reintroduce marine nutrients to farmed seafood [21,22], but would also likely reintroduce marine environmental contaminants [21]. Lipid and protein fractions (i.e., oil and meal), processed from mixed mesopelagic biomasses, are the most likely nutrient resources for formulated aquafeeds. Processing of mesopelagic catches into oil and meal alters the level of the different chemical compound groups of undesirables [21,23]. Fat soluble organic pollutants (i.e., PCDD/Fs, PCBs, PBDE) are likely to be up-concentrated in the oil fraction while the metals and metalloids (i.e., As, Cd, Hg, Cd) would increase in the meal fraction [21,23]. The fat-soluble POPs are known to readily biomagnify in an aquatic food web resulting in higher levels in organisms at a higher trophic level [24]. Harvesting mesopelagic biomass that contains lower trophic species could thus potentially lower the POP loads in farmed seafood when mesopelagic oils are used instead of pelagic fish oils.

Commercial harvest of mesopelagic biomasses used to produce feed ingredients contain different species such as jellyfish, krill, shrimps and mesopelagic fish, and the species composition can vary widely among catches [9,10]. The different species compositions of the catches partly explain the variation in metal levels among different hauls [9]. In addition, as for pelagic fish species, a seasonal and geographic variation in the levels of undesirables could be expected. The potential large variation in levels of undesirables in mixed mesopelagic biomasses means that oil and meal produced from some catches could exceed feed safety limits while for other catches not. For plant food products, processing factors databases have been established [25], which are used to assess the level of undesirables in the processed product compared to those in the harvested raw product (i.e., rapeseed oil from rapeseed). In the processing of mesopelagic biomasses into feed ingredients, such processing factors can be applied as a pro-active risk assessment tool in assessing whether or not different mixed mesopelagic catches would produce oils or meals that are in compliance with feed and food safety legislation.

The European Union (EU) has set maximum limits (MLs) for contaminants in animal feeds and feed ingredients [26]. The EU feed legislation aims to control the level of contaminants at the start of a food production chain, thus protecting consumer safety at an early stage. As novel mesopelagic feed ingredients are being investigated for possible implementation in seafood production [9,21], compliance with EU feed and food safety risk assessment is important. In studies on mixed mesopelagic biomass, catches containing mixtures of amphiphods had levels of cadmium above that allowed for feedstuffs. [9]. Other studies on individual mesopelagic species have provided theoretical calculations of the expected levels of contaminants in processed oil and meal fractions [21]. These estimates indicate high levels of undesirable trace elements such as and F in the protein fraction, frequently exceeding the MLs for feed ingredients. Despite their lower trophic level, the oil fraction of mesopelagic fish was estimated to have dioxins and furans (PCDD/Fs) levels that were above the set MLs [21]. Recently, a new risk assessment has been made on the tolerable weekly intakes (TWI) for PCDD/Fs+DL-PCBs in humans, which has been lowered from 14 to 2 pg TEQ per kg bodyweight per week [27]. Food surveillance indicates that most consumers have an intake of PCDD/Fs+DL-PCBs that exceeds the newly lowered TWI [27]. Fatty fish, including Atlantic salmon (*Salmo salar*), are one of the main sources for PCDD/Fs exposure in the adult population [27]. New proposals have been made to lower the MLs for PCDD/Fs+DL-PCBs in feed ingredients to reduce the load of these contaminants in farmed food. New feed ingredients, including mesopelagic processed ingredients, can alter the levels of PCDD/Fs+DL-PCBs in farmed seafood [19]. Feed-to-fillet transfer models in aquaculture have been developed to predict PCDD/Fs+DL-PCB levels in commercial farmed Atlantic salmon fillet when farmed on new feed ingredients [27,28]. Such models can thus aid a feed and food safety risk assessment when novel aquafeed ingredients (i.e., mesopelagic oil of meal) are exploited for the farming of seafood.

This study aims to provide a whole-chain feed and food safety assessment of undesirables in processed mesopelagic biomass catches, and in farmed seafood that is raised on feed containing this processed biomass. These undesirables include trace elements such as arsenic (As), cadmium (Cd), mercury (Hg) and lead (Pb); essential elements such as selenium (Se); and organic persistent pollutants such as dioxins (PCDD/Fs) and dioxin-like PCBs (DL-PCBs), non-dioxin-like PCBs (PCB6) polybrominated diphenyl ethers (PBDE-7), and organochlorine pesticides (OCPs). The levels of the potentially harmful substances in fishmeal and oil produced from mesopelagic biomass are compared to the levels found in commercially produced feed containing pelagic biomass (which is currently used in salmon aquaculture). Model predictions are made on the levels of PCDD/Fs+DL-PCBs in Atlantic salmon when raised on mesopelagic-based feed versus commercial feed currently used. The contaminant group of PCDD/Fs+DL-PCBs was selected as it is identified as a potential food safety risk for fatty seafood, including farmed Atlantic salmon. The predictions are based on earlier published and validated feed-to-fillet PCDD/Fs+DL-PCBs aquaculture transfer models developed for commercially produced Atlantic salmon [27,28].

## 2. Material and Methods

### 2.1. Sampling

#### 2.1.1. Biomasses Obtained from Commercial Mesopelagic Trawling

Experimental fishery for mesopelagic species was carried out in the Northern Atlantic by the 62-m-long pelagic trawler “MS Birkeland”, September to November 2019. The mesopelagic biomasses were thus obtained from one season and one general geographic area. Trawls were conducted at seven stations. Samples from four of these trawls are included in this study. Theses samples were collected at 60°04′ N 03°20′ W (station 2), 61°27′ N 01°55′ W (station 3), 62°00′ N 03°41′ W (station 6), and 59°26′ N 03°38′ W (station 7). The mean sampling depth of the trawl stations (±standard deviation) was 129 ± 30 m. Once the catch was on board, a fraction of the catch was sorted out for species identification and estimation of the catch composition. Mueller’s pearlside (*Maurolicus muelleri*) Northern krill (*M. norvegica*), and helmet jellyfish (*P. periphylla*) were the most abundant species in the catch and represented 40%, 0%, 60%; 100%, 0%, 0%; 91%, 0%, 8%; and 37%, 58%, 2% in station 2, 3, 6 and 7, respectively. For biochemical analysis, representative samples of unsorted raw material were immediately frozen at −20 °C.

#### 2.1.2. Biological Material and Processing

Frozen raw material (either a mixture of *Maurolicus muelleri* and krill or pure *Maurolicus muelleri*, see Section 2.1.1) was ground on a meat grinder immediately before use. A total of 1000 g ground raw material was added to a glass reactor with a heating jacket and overhead stirring. A total of 2.5 g (corresponding to 500 ppm tocopherols) Grindox 1032 (a water-dispersible, 20% mixed tocopherols blend in soybean oil and emulsifiers, Canisco Cultor, Braband, Denmark) and 500 g tap water (to ensure homogenous mixing and heating) were added. The mixture was heated to 85 °C with continuous stirring and kept at 85 °C for 10 min. The mixture was pressed mechanically (tincture press) through a filter cloth with a metal backing screen (not for all hauls—alternatively, the mixture was centrifuged directly and the “press liquid” decanted from the “press cake”), and the press liquid was centrifuged (20,000× *g*, 40 °C, 30 min). The supernatant was poured into a separatory funnel while the remaining sediment was combined with the press cake and homogenized in a food processor. The aqueous phase (stick-water) and the oil were separated in the separatory funnel. In the present study, a mass balance assessment was made in the amount of press-cake, oil, and stick-water produced from 1000 g mixed mesopelagic catch to which was added 500 g water. The mass balance allows investigation of the distribution of undesirables between the fishmeal and oil from a mixed mesopelagic catch. In conventional fishmeal production, the stick-water is concentrated and returned to the press-cake before drying of the mass into a “whole meal” with less than 10% moisture. In this laboratory study, this final step in meal production (stick-water returned to press-cake) was not performed. However, the concentrations of undesirables that would be present in a whole meal is calculated by adding the amounts found in the stick-water and press-cake.

#### 2.1.3. Pelagic Fish Oils and Meals Currently Used in Aquafeeds

A total of 10 randomly selected pelagic fish oils and meals were sampled at seven different aquafeed production plants in Norway. The samples were taken by the Norwegian Food Safety Authority (NFSA) in the period of the January 15, 2019 until November 25, 2019, and analyzed by the Institute of Marine Research on behalf of the Norwegian Food Safety Authority [29]. These commercially produced fish oils and meals are based on pelagic fish species from the North-Atlantic Ocean, such as blue whiting (*Micromesistius poutassou*), capelin (*Mallotus villosus*) and sandeel (*Ammodytes tobianus*). The mesopelagic oils and meals and pelagic oils and meals were analyzed with the same analytical methods (see below) [30].

### 2.2. Chemical Analysis

All methods are accredited by the Norwegian Accreditation Authority. Our laboratories participated in ring-testing organized by the European reference laboratories for the different chemical groups.

#### 2.2.1. Trace Elements

Analyses of trace elements arsenic (As), cadmium (Cd), mercury (Hg), lead (Pb), selenium (Se), cobalt (Co), copper (Cu), zinc (Zn), manganese (Mn), iron (Fe) are described in detail by Julshamn et al. [31]. Briefly, the samples were freeze-dried, homogenized, then digested in nitric acid (69% *w*/*w*) before analysis by inductively coupled plasma mass spectrometry (ICP-MS). The samples (~0.2 g) were digested in 2.0 mL of nitric acid, 130 mL Milli-Q^®^ water and 5 mL H_2_O_2_, in an ultrawave system (UltraWAVE, Milestone, Sorisole, Italy) and loaded on an autosampler. The concentrations of As, Cd, Hg and Pb were determined by ICP-MS (iCapQ ICP-MS, Thermo Scientific, Waltham, MA, USA) coupled to an auto sampler (FAST SC-4Q DX, Elemental Scientific, Omaha, NE, USA). The ICP-MS was tuned using a 1 ppb tuning solution B (Thermo Fisher, Waltham, MA, USA) in 2% HNO_3_ and 0.5% HCl. Data was processed with the Qtegra ICP-MS software (Thermo Scientific, version 2.10, 2018). The method is accredited (NS-EN 17025) by use of certified reference materials such as lobster hepatopancreas (TORT-3; National Research Council Canada, Ottawa, Ontario, Canada) and oyster tissue (SMR1566b; National Institute of Standards and Technology, Gaithersburg, MD, USA). The dry weight-based limit of quantification (LOQd.w.) was set to 0.005 mg/kg for Cd and Hg, 0.010 mg/kg for As and Se, 0.030 mg/kg for Pb and Mn, 0.1 mg/kg for Cu and Fe, and 0.5 mg/kg for Zn, with a standard sample size. The method is accredited according to ISO-17025 for the elements As, Cd, Hg, Pb, Zn, Se and Cu.

#### 2.2.2. Fluoride

Total fluoride was analyzed according to Malde et al. [32]. The fluorine content in 0.25 or 0.50 g sample material was analysed with a selective ion electrode (Thermo Orion ionpuls fluorine electrode, Orion 94-09, Beverly, MA, USA). The samples were dry-ashed in a muffle furnace (CSF 1100, Carbolite Furnaces, Bamford, Sheffield, England) at 550 °C with sodium hydroxide as an ashing aid. The dry-ashed samples were dissolved in distilled water (10 to 15 mL) and neutralized with hydrochloric acid and the dissolved samples were adjusted to the optimal pH range for fluoride determination (pH 5.2 to 5.4). The precision and accuracy of the method was assessed with certified reference material (i.e., oyster tissue, 1566a, NIST Gaithersburg, MD, USA).

#### 2.2.3. Crude Fat

The crude fat content was determined gravimetrically in wet homogenates by extraction with 30% isopropanol in ethyl acetate. The extract was filtered, the solvent evaporated, and the fat residue weighed. This method is accredited in accordance with ISO-EN 17025 and registered as a Norwegian Standard, NS 9402 [33].

#### 2.2.4. Determination of Dioxins, Furans, Polychlorinated Biphenyls, Organochlorine Pesticides, and Polybrominated Flame-Retardants

The methods have been validated by inter-laboratory tests using calibration materials, and references to the results of the proficiency test, assurance procedures, and quantification quality are given in Berntssen et al. [19]. The concentrations of dioxins and furans (PCDD/Fs) and non-ortho PCBs, mono-ortho PCBs and PBDE were determined by using high-resolution gas chromatography/high-resolution mass spectrometry (HRGC/HRMS) according to Berntssen et al. [34]. Briefly, the sample material was solvent-extracted by pressure (n-hexane for all other substances and 80:20 dichloromethane:n-hexane for PBDE) with a Dionex ASE 300 solvent extractor (Dionex, Sunyvale, CA, USA). For the online cleanup of NDL-PCBs and PBDEs, acid-impregnated silica was added to the extraction cell. Co-extracted fat was removed by adding concentrated sulfuric acid. Prior to extraction, surrogate internal standards were added (PBDE 139 EO-5100 for PBDEs, ^13^C labeled EDF-4147, 4097, 5999, 6999, 7999, 8999, 9999-3-4, 9999 for PCDD/F, PCB-53 for NDL-PCBs, and EC-4935, 4979, 4937, 4976-3, 4976, for DL-PCBs, Cambridge Isotope Laboratories, Andover, MA, USA). For PCDD/F and DL-PCBs, extracts were purified using H_2_SO_4_ on silica, multilayered silica, basic alumina and carbon columns, respectively (FMS, Waltham, MA, USA; for solvent conditions, see [18]). The samples were concentrated by pressurized evaporation (Turbovap II™ Zymark, Hopkinton, MA, USA). A ^13^C labeled performance standards mixture (EC-4979 for DL-PCBs and EDF 5999 for PCDD/F, Cambridge Isotope Laboratories, Andover, MA, USA) was used prior to analyses by HRGC/HRMS (MAT 95XL Thermo Finnigan, Bremen, Germany) using a fused silica capillary column (30 m × 0.25 mm, i.d. and 0.25 μm film thickness, RTX-5SILMS, Restek, Bellefonte, PA, USA). Quantification was performed according to the internal standard isotope dilution method using congener-specific relative response factors (RRFs) determined from three-point calibration standard runs (CS1–CS3, Cambridge Isotope Laboratories, Andover, MA, USA), USEPA 1613 method [35]. Recovery values were between 78 and 110% and PCCD/F and DL-PCBs values are expressed as pg upper bound WHO-TEQ g^−1^wet weight (w.w.) using the WHO-TEFs from 2005. For OCPs, extracts were purified in an automated column system (ASPEC™ XL4, Gilson, Winfield, PA, USA) with three sequential solid-phase extraction (SPE) columns (Chem Elut™, BondElut^®^ C18, and BondElut^®^ Florisil columns, in that order, Varian Inc., Palo Alto, CA, USA. For solvent conditions, see [19]). The levels of dichlorodiphenyltrichloroethane (DDT) are expressed as the molecular weight-corrected ortho- and para-forms of DDT and its metabolites dichlorodiphenyldichloroethylene (DDE) and dichlorodiphenyldichloroethane (DDD). The levels of chlordane are the summation of oxy-chlordane, trans-chlordane, and cis-chlordane. The levels of hexachlorocyclohexane (HCH) are the summation of α, β, γ-HCH. The levels of dieldrin are the sum of aldrin and dieldrin. Determination of indicator non-dioxin-like PCBs (NDL-PCBs) are expressed as the summation of PCB-28, -52, -101, -138, -153, -180 (PCB-6). The NDL-PCBs and OCPs were analyzed by gas chromatography/mass spectrometry (TRACE GC Ultra™/DSQ™ Single Quadrupole GC/MS, Thermo Finnigan, Bremen, Germany) with a fused silica capillary column (30 m × 25 mm i.d. 25 μm film thickness HP-5MS Column, Agilent J&W, Sanata Clara, CA, USA). For PBDEs, the on-line purified extracts (by acid-impregnated silica as a fat retainer in the accelerated solvent extractor extracts) were analyzed by gas chromatography/mass spectrometry (TRACE GC Ultra™/DSQ™ Single Quadrupole GC/MS, Thermo Finnigan, Bremen, Germany) equipped with an RTX-5MS capillary column (30 m × 0.25 mm i.d. 25 μm film thickness, Restek, Bellefonte, PA, USA). PBDE levels are expressed as the summation of PBDE-28, -47, -99, -100, -153, -154, -183 (PBDE-7). Quantification was according to the internal standard (IS) method using congener-specific RRFs from a three-point linear congener-specific external standard curve relative to the internal surrogate standard. Recovery was evaluated for each congener by spiking each sample matrix with internal standards for all congeners at three levels. The recovery of NDL-PCBs and OCP was between 85 and 110% and recovery for PBDE congeners was between 81 and 118%. Samples were run in batches of 12, with one procedural blank, one in-house performance evaluation standard (homogenized salmon fillet) and 10 samples with a duplicate of the last sample. The limit of quantification was determined for each determination and congener by using nine times the noise level (three times the limit of detection). The limit of detection (LOD) was statistically estimated as the analyte concentration giving a peak signal of three times the background noise from an internal-surrogate standard-spiked procedural blank. The LOD was calculated using a software option for estimating the signal-to-noise (S/N) ratio and referring this value to an S/N value of three. Trueness was set to −2.0 ≤ z-score ≤ 2.0 and repeatability as RSD (%) > 10 and better by using calibration material and spiked samples. The analytical methods have been validated by inter-laboratory tests using calibration materials, references to the results of the proficiency test, quantification quality, and assurance procedures given by Berntssen et al. [19]. The legislation for the PCDD/Fs+DL-PCBs in feed and food is based on the summation of 29 different congeners expressed as upper bound (UB) values in which undetected congeners are expressed as their limit of quantification (LOQ). When concentrations are expressed as lower bound (LB) values, the congeners that are not detected are set zero. As the concentrations for contaminant groups that consist of the summation of several congeners (PCDD/Fs, DL-PCBs, PBDE-7, PCB-6, sum chlordane, sum HCH, sum DDT, sum aldrin+dieldrin) would strongly depend on the number of congeners detected as well as their matrix specific LOQ, all POP values are expressed as UB and LB in the present study.

### 2.3. Processing Factors

Processing factors (PF) are expressed as concentration in mesopelagic oil or meal (WW) per concentration in the raw mesopelagic biomass (WW).

The processing factors were expressed as:

$$\mathrm{PF}=\frac{C_{\mathrm{Oil}\;\mathrm{or}\;\mathrm{meal}\;\left(\mathrm{WW}\right)}^{}}{C_{\mathrm{mesopelagic}\;\mathrm{biomass}\;\left(\mathrm{WW}\right)}^{}},$$

### 2.4. Predictions of PCDD/Fs+DL-PCBs in Atlantic Salmon Farmed on Mesopelagic Oil and Meal

A simple one-compartment toxicokinetic feed-to-fillet transfer model was used, as published and validated earlier [19]. The transfer model is derived from a fish biomagnification model as described by Sijm et al. 1992 [36] and based on the congener specific uptake rates (α, ng WHO2005-TEQs day^−^^1^) of the 29 different congeners of the PCDD/Fs and DL-PCBs that are included in the legislation on dioxins and dioxin-like PCBs [27,28]. In addition to the uptake rates, the model includes elimination rates (K, day^−1^), growth dilution (γ, % body weight day^−^^1^), feeding rate (F, %body weight day^−^^1^), and initial concentration in the fish fillet (C_fi__sh0_, ng WHO-TEQs kg^−^^1^).

$$C_{\mathrm{fish}}^{}\left(t\right)=\frac{\alpha \;F\;t}{K+\gamma }\;\;{\;C}_{\mathrm{feed}}^{}\;\left(1-e_{}^{-\left(K+\gamma \right)t}\right)+C_{\mathrm{fish}0}^{-\left(K+\gamma \right)t}$$

The model allows simulation of the long term (>one year) feed to-fillet transfer of POPs in Atlantic salmon using realistic farming conditions such as feed intake and growth rates, which determine final PCDD/Fs+DL-PCBs levels in farmed fish [19]. To simulate a standard commercial production of Atlantic salmon over time (t), a growth rate of 0.64% day^−1^ and a feeding rate of 0.78% BW day^−1^ was used with a farming time of 13 months with an expected market-sized weight of 4.5 kg [19]. The levels of PCDD/Fs+DL-PCBs in mesopelagic or pelagic aquafeeds are based on the currently used salmon feed formulation with marine oil inclusion of about 10% and marine protein inclusion of 15% [37]. PCDD/Fs+DL-PCBs in aquafeeds were based on the standard aquafeed formulation [37], earlier analysed levels in plant-feed ingredients [19], and the level of PCDD/Fs and DL-PCBs in mesopelagic and pelagic oil and meal as reported in this study (see above).

## 3. Results and Discussion

### 3.1. Essential Minerals

The levels of essential trace-elements in the mixed mesopelagic biomasses and processed mesopelagic meal are shown in Table 1. Concentrations are presented on a dry weight (DW) basis as the biomass water content varies among the different hauls. The element levels in the meal are a mass-balance summation of the DW levels analyzed in the press-cake and stick-water. All the water-soluble trace-elements were detected in the stick-water, which contributed to the final levels in mesopelagic meal (meal = press-cake + stick-water). Adding the stick water back to the press-cake increased the levels of F, Co, Se, Mo with 39, 21, 15, and 14%, respectively, while stick-water contributed little (<2%) to the final mesopelagic meal levels of Cu, Zn, Mn and Fe. In formulated aquafeeds, these essential trace elements, besides F, are supplemented in the form of a mineral mixture to cover the nutritional requirement of the farmed fish [18,29]. The natural background levels of trace elements in processed mesopelagic meal were compared with the levels analyzed in the monitoring program of commercial pelagic fish meal on the Norwegian market [29] (Table 1). The processed mesopelagic meal has similar levels of essential trace-elements as commercial pelagic fish meal on the Norwegian market, indicating that similar mineral mixes can be used in aquafeeds based on mesopelagic-based meals compared to conventional commercial meals. An exception is iron (Fe), of which lower levels were found in the mesopelagic meal compared to the commercial pelagic meals. Iron is one of the minerals commonly supplemented in the mineral mixture [38]. If mesopelagic meals are used instead of conventional pelagic meals, an increase in Fe mineral supplementation is needed if the level of this micronutrient is to be maintained in aquafeeds.

The MLs for undesirables in feed and feed ingredients are set on an 88% dry matter basis and the levels in the mesopelagic meals are thus given for the same dry matter content for comparison with legislative limits [39] (Table 1). For all essential trace elements supplemented to aquafeeds (Fe, Zn, Cu, Mn, Co, Mo), the upper limits are set on the final feeds and not feed ingredients through the Legislation on feed additives (EC) No 1831/2003). Fluoride (F) is an essential element which is not supplemented to aquafeeds, and MLs are set for feed ingredients in addition to the final feeds. The level of F in processed mesopelagic meals in the present study had a large variation, depending on the relative amounts of krill in the mixed catches, with mean levels of 853 mg kg^−1^ (min.–max. 93–1804 mg kg^−1^). The mean F levels in the mixed mesopelagic meals are thus higher than the ML set for F in feed materials of 500 mg kg^−1^ [40] (Table 1). The exoskeleton of marine crustaceans are well-known to contain high F levels, causing concentrations in mesopelagic catch crustaceans to vary from 2700 to 3700 mg kg^−1^ DW for species such as *Meganyctiphanes norvegica* and 60 to 360 mg kg^−1^ DW for *Periphylla*, *Eusergestes arcticus* and *Pasiphaea spp* [21]. The ML for feed ingredients could limit the use of two out of four mesopelagic meals produced from the mixed mesopelagic catches in the present study. However, the ML for feed ingredients based on marine crustaceans such as krill is much higher and was set to 3000 mg kg^−1^ and none of the mesopelagic meals in this study exceeded this limit [40]. It is unclear whether meal produced from mixed mesopelagic biomass from the present study falls under the general ML for feed materials of animal origin or ML for feed materials of marine crustaceans. Several studies with krill in farmed fish species have showed that the F in krill or amphipods meals does not elevate the muscle F level, and thus would not likely form a food safety risk [41]. However, in order to reduce the F load in krill meals for human consumption as well as aquafeed ingredients, several approaches have been investigated, such as chemical extraction [42,43], deshelling [44,45], or the use of calcium feeds supplement to reduce the bioavailability of F from krill meals used in the aquafeeds [46]. Supplementation with Ca could alter the bioavailability of other trace elements as Ca levels in aquafeeds are known to reduce the bioavailability of other essential trace elements such as Zn [47] and Mg, and Fe [38].

### 3.2. Non-Essential Metals and Metalloids

The levels of non-essential metals and metalloids (As, Pb, Cd, Hg) in the mixed mesopelagic biomasses and processed mesopelagic meal are given in Table 2. As for the essential trace elements, stick-water contributed to the final mesopelagic levels of water-soluble non-essential elements (As, Cd, Pb, Hg). Stick-water contributed to 38% of the As and 14% of Hg, while stick-water contributed little (<2%) to the final levels of Cd and Pb. None of these non-essential trace elements exceeded the ML for these undesirables in fish meals (Table 2).

The Cd levels in mesopelagic meals were higher than in commercial pelagic fish meals surveyed on the Norwegian market [29] (Table 2). Earlier studies on mixed mesopelagic biomass intended for aquafeeds showed a large variation in Cd among different catches from inner fjord systems or open waters off the south-west coast of Norway [9]. Especially catches from open waters that contained amphipods resulted in cadmium levels exceeding the MLs in feedstuffs [9]. However, these mixed mesopelagic biomasses were not processed into meal as performed in this study [9]. Earlier studies on theoretical calculations on meal produced from single mesopelagic species also reported relative high Cd levels [21,23]. In general, internal organs such as liver, intestine, and especially kidney have natural high background levels of Cd, which are often around three to fivefold higher than background levels reported in muscle [48,49]. A relatively high viscera to muscle ratio for small (3 to 8 cm) mesopelagic fish species (i.e., *Maurolicus muelleri* and *Benthosema glaciale* [9,22]) compared to larger (13 to 31cm) pelagic fish species (i.e., *Engraulis ringens* or *Micromesistius poutassou* [50,51]) could partly explain the higher Cd levels in meals produced from mesopelagic fish compared to meals produced from pelagic fish. In contrast to Cd, Pb is more equally distributed among the internal organs and muscle, while bone structure carries most of the background Pb levels [52]. The Pb levels in mesopelagic meal are similar to that of pelagic fish meal (Table 2). As opposed to Cd, the Hg levels in mesopelagic meals were lower than those seen in commercial pelagic fish meals (Table 2). Mercury in the marine ecosystem is mostly in the organic form (methylmercury) which is known to biomagnify in the marine food web, resulting in increased levels in organisms at higher trophic levels [53]. The biomagnification of methylmercury is reflected in the lower Hg levels in the lower trophic mesopelagic meal compared to higher trophic pelagic meal.

Total As levels in the mesopelagic meals were in the same range as in commercial pelagic fish meal (Table 2). The total As levels are 10- to 100-fold higher than the other non-essential trace elements such as, for example, Cd, Pb, and Hg (Table 2). Marine fish contain relatively high levels of total arsenic, which is mostly in organic forms, such as arsenobetaine [54]. These organic As forms can be both water-soluble and lipid-soluble and they have different extent in methylation and oxidation states [55]. Marine organisms have a wider range of organic As forms compared to terrestrial samples, where the predominant forms are inorganic As and simple methylated organic forms [56]. The toxicity of As depends on the form, where inorganic As is known to be toxic, and the main As form that is risk-assessed in feed and food products [57]. Within the EU, MLs are established for total As in feed and feed ingredients, whereas the regulations in food are limited [58]. Organic forms such as arsenobetaine are considered to be non-toxic to humans [59]. The ML for As in feed materials of marine origin are higher (25 mg/kg) than that of feed materials of plant origin (2 and 4 mg/kg), and the justification for the higher MLs for marine feed and feed materials is that organic As does not pose a risk because of its lower toxicity compared to inorganic As [58,59]. However, an overview on recent studies on arsenic species in marine products showed that some organoarsenicals or their metabolites are cytotoxic, similar to inorganic arsenic, stressing the need for further development of analytic methods and toxic assessment of these marine organic arsenic forms [60]. In the present study, arsenic was the only trace element that was also detected at relatively high levels in the processed mesopelagic oils, with median levels of 6.3 mg kg^−1^ (min.–max. 4.4–8.3 mg kg^−1^). The levels in oils are nearly the same as in the mesopelagic meals (median 6.0, min.–max. 5.1–7.8 mg kg^−1^) (Table 2). The high As levels in marine oils are dominantly in organic arseno-lipid forms [61]. As opposed to arsenobetaine, less is known on their potential toxicity compared to the known toxic inorganic arsenic forms [60]. However, in vitro and model organism studies indicate the potential toxicity of arsenolipids and their metabolites [62,63,64], and hence, the need for a full hazard identification of subclasses of arsenolipids in order to assess ML for total As in marine oils.

### 3.3. Organic Pollutants (POPs) in Mesopelagic Oils and Meals

The level of POP in oils processed from mesopelagic biomasses are given in Table 3. The European legislation for PCDD/Fs+DL-PCBs in feed and food is based on upper bound (UB) values, which means that when contaminants are not detected, the limit of quantification (LOQ) for the specific contaminant is presented. In Table 3, the lower bound (LB) levels are also presented, in which undetectable contaminants are set to zero. Large differences were seen between PCDD/Fs levels expressed as UB or LB (1.4 and 0.5 WHO-TEQ pg g^−1^, respectively) (Table 3). The concentrations for PCDD/Fs and DL-PCBs are based on the summation of 29 different congeners (seven for PCDD, 10 for PCDF, 12 for DL-PCBs). The detection, or non-detection, of the 29 congeners will affect concentrations of PCDD/Fs+DL-PCBs expressed as UB and LB [28]. In the present study, none of the 10 PCDF congeners and only four out of seven PCDD congeners were detected in the mesopelagic oils. The reported UB for total PCDD/F levels are thus a summation of 13 different LOQs for the undetected congeners, accounting for 64% of the reported UB levels. Expressing PCDD/Fs as UB would, hence, overestimate the actual level present. For DL-PBs, 16 out of 17 DL-PCB congeners were detected, and the difference between UB and LB DL-PCBs is thus less than for PCDD/Fs (0.74 vs 0.73, WHO-TEQ pg g^−1^, respectively) (Table 3).

Despite the relatively large contribution of analytical LOQ, the UB levels of PCDD/F+DL-PCBs, PCB-6, PBDE-7, and OCP in mesopelagic oil were lower than those seen in commercial oils from pelagic fish used in Norwegian aquafeeds. For example, for all POPs, the highest analyzed UB levels in the four mesopelagic oils were still lower than the reported mean level for commercial pelagic fish oils (Table 3). The POPs are persistent fat-soluble environmental pollutants that are globally dispersed and readily biomagnify in the marine food web, with increasing levels at higher trophic levels [24]. The commercial fish oils currently used are produced from pelagic fish species such as blue whiting, capelin (*Mallotus villosus*) and sandeel (*Ammodytes tobianus*) and their higher trophic level compared to the lower trophic mesopelagic biomasses harvested in this study (including jellyfish and crustaceans) could at least partly explain the lower POP levels observed in mesopelagic oil compared to pelagic fish oil. The levels of POPs in commercial pelagic fish oils vary widely depending on season and geographical origin of the pelagic catch [65,66]. Fish oils from pelagic fish species from the Pacific Ocean generally contain lower PCCD/F levels and to a lesser degree lower DL-PCBs levels than fish oils produced from fish of Atlantic Ocean origin [65,67]. Fish oils of Baltic sea origin contain relatively high levels of both PCCD/Fs and DL-PCBs [67], and Baltic fish oils are often decontaminated in order to comply with the MLs for PCDD/Fs and DL-PCBs in fish oils [34]. Furthermore, during early spring, when the fat content decreases in feral fish, the concentration of PCDD/Fs and PCBs increases in the extracted oil of blue whiting [66]. Additionally, for mesopelagic biomasses, seasonal variations could be expected. For example, different feeding behavior through seasons might affect the level of undesirables in *Maurolicus muelleri*. This species mainly feeds on copepods in autumn and winter, whereas in early spring, diatoms are incorporated in their feeding regime [68], and the type of prey is of importance in the metal accumulation in marine fish [69]. However, in mixed catches, Olsen et al. [9] did not find a clear relationship between the gross chemical composition and season of catch. The species composition was the most important factor determining the chemical composition of the catch [9]. Future surveillance of mesopelagic oils will reveal if there is significant seasonal variation in undesirables. Earlier theoretical estimations on POP levels in mesopelagic oils (based on analyzed levels in individual mesopelagic fish species) predicted PCDD/Fs levels above the permitted levels for feed ingredients [21]. In contrast, the present study showed that the levels of PCDD/Fs and DL-PCBs in produced mesopelagic oils were below the set MLs for all POPs, and were in general lower than the pelagic fish oils currently used in commercial salmon feeds.

Table 4 gives the levels of POPs in mesopelagic raw biomass and the thereof processed meal. None of the fat-soluble POPs were detected in the stick-water as opposed to the press-cake that still contained some rest-lipids (9 to 14% of WW) after the production of the oil fraction. The POPs in stick-water are, hence, solely based on the analytical LOQ levels. In order not to overestimate the POP levels in meal (press-cake+stick-water), stick-water LB levels were used in the summation of press-cake+stick-water to give levels in mesopelagic meals. As for the levels of POPs in the mesopelagic oils versus pelagic oils (Table 3), the POP levels in mesopelagic meal were similarly lower than those observed in commercial pelagic fish meals (Table 4). PCDD/Fs, chlordane, and aldrin+dieldrin were an exception (Table 4): the levels were similar or higher than those observed in surveyed pelagic meals (Table 4). However, the large difference in UB and LB levels (e.g., 0.49 and 0.01 WHO-TEQ pg g^−1^ for PCDD/Fs, respectively) indicate that the UB levels are mostly driven by a summation of LOQ rather than quantified levels [28].

### 3.4. Processing Factors

Table 5 lists the processing factors for POPs and trace elements in mesopelagic oil and meal, expressing the up-concentration (factor > 1) or dilution (factor < 1) of these contaminants when mesopelagic mixed biomass catches (DW) are processed into mesopelagic oil or meal aquafeed ingredients (median, min.–max, n = 4). As for the POPs concentrations in oils (Table 3), the processing factors for POP were expressed as upper-bound (UB) or lower bound (LB). Especially for contaminant groups where few congeners were detected (e.g., sum PCDD/Fs and sum DDT), a large difference in UB or LB processing factors was seen as opposed to the processing factors for contaminants where all congeners were detected (e.g., DL-PCBs and PCB-6).

The highest processing factors for POPs in the production of mesopelagic oils were seen for PCDD/Fs+DL-PCBs, PCB-6, PBDE, HCB, HCH and sum aldrin dieldrin (~4 to 5), while chlordane had lower processing factors (~1) (Table 5). The processing factors for POPs in mesopelagic meal are lower compared to oil varying from ~0.7 (PCB-6) to ~0.2 (PBDE-7 and PCDD/Fs). For trace elements, the processing factors for producing mesopelagic meal varied from ~1 to 2 (Table 5). Fluoride was also the trace element with the highest variation in processing factors (min.–max. 1–5), which strongly depends on the F level in the raw biomass and, hence, the number of crustaceans in the marine mesopelagic mixed biomass catch. The processing factors for F can be used to predict the F levels in mesopelagic meals when the catch contains a relative high portion of krill. Arsenic was the only trace element that was transferred from mesopelagic biomass to oil. The processing factor of ~0.6 reflects the relatively high presence of arsenolipids in the mesopelagic biomass, which is similar for what is reported for pelagic fish [61], thus highlighting the need for analytical identification and toxicological assessment of these marine As forms [62,63,64].

### 3.5. Predicting Levels of PCDD/F+DL-PCBs in Farmed Seafood Raised on Mesopelagic Oils

Recently, a new risk assessment has been performed for animal and human health related to the presence of PCDD/Fs and DL-PCBs in feed and food [27]. Based on new risk assessment on the fertility of young men following pre- and postnatal PCDD/Fs and DL-PCBs exposure, the tolerable weekly intake (TWI) has been lowered from to 14 to 2 pg TEQ/kg bw/week [27]. With occurrence and consumption data from European countries, the estimated mean and high PCDD/Fs and DL-PCBs intake considerably exceeded the newly established TWI. Fatty fish is one of the main contributors, in addition to dairy products, to the total dietary intake of PCDD/Fs and DL-PCBs among the European consumers [27]. Following these new intake recommendations, action has also been initiated to re-evaluate the ML for PCDD/Fs+DL-PCBs in oils and meal used in animal feeds, in order to lower the PCDD/Fs+DL-PCBs levels in farmed seafood. The lower POP loads in mesopelagic oils compared to pelagic oil are likely to give farmed seafood with lower levels of POPs in the fillets. Validated toxicokinetic feed-to-fillet transfer models have been developed for the aquaculture of seafood such as Atlantic salmon, enabling the prediction of fillet PCDD/F and DL-PCBs concentrations from known feed concentrations based on the congener specific uptake and elimination rates [27,28]. In addition, these models include variations in aquacultural factors such as growth and feed intake, which are important additional factors in determining the final PCDD/F+DL-PCBs levels in farmed seafood [27,28]. Using these earlier published transfer models, fillet PCDD/Fs+DL-PCBs levels predictions were made when mesopelagic oils are being used instead of pelagic fish oils. Input data included a realistic commercial feed intake and growth rates [28] and levels of PCDD/Fs+DL-PCBs in mesopelagic or pelagic oils (Table 3). For pelagic or mesopelagic oils, an aquafeed formulation level of 10% was used as is currently used in Norwegian salmon feed [37]. Model predicted fillet levels for salmon reared on aquafeeds based on commercial pelagic oil levels (Table 3) was 0.54 WHO-TEQ pg g^−1^, which is similar to the levels currently reported in the fillets of commercially produced Atlantic salmon in Norway (0.52 WHO-TEQ pg g^−1^ ww). When replacing pelagic oils with mesopelagic oils in a similar feed formulation, the predicted farmed Atlantic salmon fillet levels were reduced to 0.39 WHO-TEQ pg g^−1^ ww. Although the PCDD/Fs+DL-PCBs levels in mesopelagic oils were nearly half (~50%) of the levels in pelagic oils, the use of mesopelagic oil in salmon farming only reduced the fillet levels by ~30%, because other feed ingredients such as plant oils and fish meal also contribute to the PCDD/Fs+DL-PCBs load in the aquafeeds [28].

## 4. Conclusions

The present study provides a whole-chain feed and food safety assessment of mesopelagic biomasses processed into feed materials and used in the farming of seafood. The study showed that mesopelagic oil processed from different commercial mixed mesopelagic catches had levels of POPs which were in general lower than those observed in commercial pelagic fish oils currently used in fish feeds in Norway. All reported levels were under the set EU MLs for these undesirables in aquafeed oils. For the trace elements, Cd had a relative high level compared to pelagic fish meals but not exceeding the set MLs in fish meals. Arsenic levels were similarly high in mesopelagic biomass and in the produced meal and oil (as was found for pelagic fish oils and meals). The total As for meals was under the MLs set for fish meals and oils used in fish feeds. There is a need for analytical identification and toxicological assessment of organic As forms, including arsenolipids present in marine oils. Fluoride was the only trace element that exceeded the general ML for feed ingredients, but the specific ML for feed ingredients produced from crustaceans was not exceeded. There was a large variation in the F level in the mixed biomass depending on the fraction of marine crustaceans (i.e., krill) present in the catch. The study provides processing factors that can be used to predict levels of POPs, metals and metalloids in oils and meals produced from mixed mesopelagic biomass catch. The F processing factor can be used to estimate the maximum fraction of krill that can be included to produce meal with an acceptable F concentration. In the present study, only mesopelagic biomasses from one geographic area (north-east Atlantic) and one season (fall-winter) was processed. As for pelagic oils and meals, more information is needed on regional and seasonal variations of contaminants in mesopelagic produced feed ingredients. The use of mesopelagic oils instead of pelagic oils is likely to lower the PCDD/Fs+DL-PCBs load in the fillets of farmed fatty seafood such as Atlantic salmon by 30%. Despite the apparent low risk for feed or food safety of processed mesopelagic biomasses to be used in aquaculture, more knowledge is needed on the mesopelagic stocks and their dynamics and how they contribute to the global CO_2_ budget. A commercial harvesting would require proper integrated stock assessments in order to fish at a sustainable level not harming the marine ecosystem or the global CO_2_ budget and not facilitate climate change [12].

## Figures and Tables

**Table 1:** Concentrations of the essential trace elements, iron (Fe), selenium (Se), manganese (Mn), zinc (Zn), copper (Cu), cobalt (Co), molybdenum (Mo), and fluoride (F) in mg kg^−1^ dry weight (DW) (mean, (minimum-maximum), n = 4) in the raw mesopelagic biomass, the concentration in processed mesopelagic meal (DW), commercial pelagic fish meal from the monitoring program (n = 9), and mesopelagic meal expressed as 88% DW. For comparison, the maximum levels (ML) given in EU Directive 2002/32/EC and maximum content given in the Register of Feed additives ((EC) 1831/2003) are shown. * ML for marine crustaceans, ** feed for Salmonids.

Compound	Processing		Commercial Samples	Comparison to Legislation		
	Mesopelagic Biomass	Mesopelagic Meal	Pelagic Fish Meal	Mesopelagic Meal 88%DW	Feed Material	Animal Feed
	Mean	Mean	Mean	Mean		
	(min–max)	(min–max)	(min–max)	(min–max)	ML	ML
Fe	46	80	171	70		750
	(38–61)	(70–94)	(52–470)	(62–82)		
Se	2.3	2.7	2.5	2.6		0.7
	(1.4–3.1)	(2.1–3.9)	(1.7–3.8)	(2.1–3.8)		
Mn	2.8	5.0	5.7	4.42		100
	(1.8–3.5)	(3.4–6.8)	(2.5–10)	(3.0–5.9)		
Zn	35	63	68	55		180 **
	(29–43)	(57–70)	(52–81)	(50–62)		
Cu	7.6	11	6.2	9.9		25
	(2.5–18)	(3.63–28)	(2.6–26)	(3.2–25)		
Co	0.053	0.035	0.05	0.031		1
	(0.035–0.074)	(0–0.076)	(0.02–0.08)	(0–0.055)		
Mo	0.15	0.15	0.23	0.13		2.5
	(0–0.23)	(0.02–0.23)	(0.18–3.8)	(0.018–0.20)		
F	433	863	NA	853	500/3000 *	350
	(17–1157)	(92–1804)	NA	(92–1782)		

**Table 2:** Concentrations of the trace elements, arsenic (As), cadmium (Cd), mercury (Hg), and lead (Pb) in mg kg^−1^ dry weight (DW) (mean, (minimum–maximum), n = 4) in the raw mesopelagic biomass the concentration in processed mesopelagic meal (DW), commercial pelagic fish meals (n = 9), and mesopelagic meal expressed as 88% DW. For comparison, the maximum limits (ML) given in EU Directive 2002/32/EC is given for fish meals and animal feeds.

	Processing		Surveillance of Commercial Samples	Comparison to Legislation		
	Mesopelagic Biomass	Mesopelagic Meal	Pelagic Fish Meal	Mesopelagic Meal	Fish Meal	Animal Feed
	Mean		Mean	Mean 88%DW	ML	ML
	(min–max)		(min–max)	(min–max)		
As	9.6	9.7	7.3	8.6	25	10
	(7.2–13)	(5.8–12)	(2.6–12)	(5.1–10)		
Cd	0.61	0.79	0.47	0.68	2	1
	(0.1–1.3)	(0.24–1.9)	(0.12–1.0)	(0.19–1.7)		
Hg	0.028	0.05	0.13	0.05	0.5	0.2
	(0.016–0.046)	(0.016–0.15)	(0.02–0.19)	(0.014–0.13)		
Pb	0.05	0.06	0.08	0.06	10	5
	(0.016–0.046)	(0–0.13)	(<0.005–0.13)	(0–0.12)		

**Table 3:** Concentrations of dioxins (PCDD/F), dioxin-like PCBs (DL-PCBs) (WHO-TEQ pg g-1 per DW), polybrominated diphenyl ether mixtures (PBDEs) and organochlorine pesticides (ng g^−1^ per DW) (mean, minimum–maximum, n = 4) in the raw mesopelagic biomass, the concentration in processed mesopelagic oil, commercial pelagic fish oils (n = 9). Concentrations are expressed as upper-bound (UB) with undetected congeners expressed as limit of quantification or lower-bound (LB) with undetected congeners expressed as zero. For comparison, the maximum residue level (ML) based on upper-bound concentrations, given in EU Directive 2002/32/EC, are shown.

Compounds	Processing	Surveillance of Commercial Samples	Comparison to Legislation	
	Mesopelagic Oil	Pelagic Fish Oil	Fish Oil	Animal Feed
	Mean	Mean		
	(min–max)	(min–max)	ML	ML
Sum (PCDD+PCDF) (UB)	1.4	1.6	5.00	1.75
	(1.1–1.6)	(0.9–3.2)		
Sum (PCDD+PCDF) (LB)	0.50			
	(0.28–0.71)			
DL-PCBs (UB)	0.74	2.4		
	(0.39–0.90)	(0.4–5.1)		
DL-PCBs (LB)	0.73			
	(0.38–0.89)			
Sum PCDD/F Dl-PCBs (UB)	2.1	4.0	20	6
	(1.5–2.4)	(1.0–8.0)		
Sum PCDD/F Dl-PCBs (LB)	1.2			
	(0.66–1.6)			
PCB-6 UB	8.9	37	175	40
	(6.1–12)	(3.0–79)		
PCB-6 LB	8.9			
	(6.1–12)			
PBDE (UB)	1.4	4.1		
	(0.83–2.3)	(0.8–9.0)		
PBDE(LB)	1.2			
	(0.54–2.3)			
HCB (UB)	5.3	7.5	200	10
	(3.3–7.0)	(1.2–21)		
HCB (LB)	5.33			
	(3.3–7.0)			
sum DDT/E/D (UB)	8.4	40	500	50
	(6.1–11)	(9.0–73)		
sum DDT/E/D (LB)	6.8			
	(3.8–11)			
sum HCH (UB)	1.1	0.92		
	(1–1.3)	(0.0–3.3)		
sum HCH (LB)	0.65			
	(0.50–0.80)			
sun aldrin dieldrin (UB)	8.3	8.50	100	20
	(6.3–10)	(0.6–15)		
sun aldrin dieldrin (LB)	8.0			
	(5.2–10)			
chlordane (UB)	2.9	5.90	50	20
	(2.3–3.3)	(1.5–17)		
chlordane (LB)	0.26			
	(0–0.39)			

**Table 4:** Concentrations of dioxins (PCDD/F), dioxin-like PCBs (DL-PCBs) (WHO-TEQ pg g^−1^ per DW), polybrominated diphenyl ether mixtures (PBDEs) and organochlorine pesticides (ng g^−1^ per DW) (mean, minimum-maximum, n = 4) in the raw mesopelagic biomass, the concentration in processed mesopelagic meal (DW), surveyed commercial pelagic fish meals (n = 9), and mesopelagic meal expressed as 88% DW. Concentrations are expressed as upper-bound (UB) with none detected congeners expressed as limit of quantification or lower-bound (LB) with undetected congeners expressed as zero. For comparison, the maximum limit (ML) based on upper-bound concentrations, given in EU Directive 2002/32/EC are shown.

Compound.	Processing		Surveillance of Commercial Samples	Comparison to Legislation		
	Mesopelagic Biomass	Mesopelagic Meal	Pelagic Fish Meal	Mesopelagic Meal 88% DW	Fish Meal	Animal Feed
	Mean		Mean	Mean		
	(min–max)		(min–max)	(min–max)	ML	ML
Sum (PCDD+PCDF) (UB)	0.64	0.49	0.33	0.43	1.25	1.75
	(0.49–0.80)	(0.38–0.55)	(0.26–0.42)	(0.39–0.48)		
Sum (PCDD+PCDF) (LB)	0.11	0.01		0.01		
	(0.05–0.17)	(0–0.04)		(0–0.02)		
DL-PCBs (UB)	0.19	0.13	0.50	0.12		
	(0.11–0.29)	(0.08–0.18)	(0.08–0.8)	(0.07–0.15)		
DL-PCBs (LB)	0.19	0.022		0.021		
	(0.11–0.29)	(0.001–0.06)		(0–0.059)		
Sum PCDD/Fs+DL-PCBs (UB)	0.83	0.64	0.80	0.54	4	6
	(0.64–1.0)	0.46–0.73	(0.4–1.2)	(0.41–0.64)		
Sum PCDD/Fs+DL-PCBs (LB)	0.3	0.10		0.089		
	(0.16–0.42)	(0.01–0.22)		(0–0.1)		
PCB-6 UB	2.4	2.2	4.7	2.0	30	40
	(1.7–3.7)	(0.85–1.6)	(0.6–8.7)	(1.3–2.8)		
PCB-6 LB	2.4	1.7		1.5		
	(1.6–3.7)	(0.86–3.0)		(0.75–2.6)		
PBDE (UB)	0.33	0.48	0.52	0.42		
	(0.24–0.48)	(0.45–0.54)	(0.06–1.0)	(0.40–0.47)		
PBDE(LB)	0.26	0.06		0.05		
	(0.12–0.43)	(0–0.23)		(0–0.20)		
HCB (UB)	1.6	1.4	2.4	1.2	10	10
	(1.3–2.2)	(1.0–1.8)	(1.2–3.3)	(0.91–1.6)		
HCB (LB)	1.6	1.4		1.2		
	(1.3–2.2)	(1.4–1.8)		(0.91–1.6)		
Sum DDT/E/D (UB)	3.4	1.8	6.8	1.6	50	50
	(2.7–4.2)	(1.0–3.5)	(0.6–13.3)	(0.91–3.1)		
Sum DDT/E/D (LB)	1.1	1.2		1.1		
	(0.94–2.8)	(0.62–2.3)		(0.55–2.1)		
Sum HCH (UB)	2.4	1.4		1.2		
	(1.6–3.5)	(1.3–1.5)		(1.1–1.3		
Sum HCH (LB)	0.16	0.16		0.14		
	(0–0.26)	(0.10–0.21)		(0.09–0.18)		
Sum aldrin+dieldrin (UB)	2.2	1.8	1.3	1.6	10	20
	(1.2–4.2)	(0.68–2.9)	(0.2–2.0)	(0.60–2.4)		
Sum aldrin+dieldrin (LB)	1.6	0.8		0.69		
	(0.0–3.3)	(0–1.1)		(0–1.1)		
Chlordane (UB)	4.3	1.8	1.6	1.6	20	20
	(3.8–4.9)	(1.7–2.0)	(0.5–2.6)	(1.5–1.8)		
Chlordane (LB)	0.25	0.19		0.17		
	(0–0.99)	(0–0.78)		(0–0.69)		

**Table 5:** Processing factors (ratio of wet weight-based contaminant concentrations in raw mesopelagic biomass to produced oil or meal on dry weight (DW) basis) of dioxins (PCDD/F), dioxin-like PCBs (DL-PCBs) (WHO-TEQ pg g^−1^ DW), polybrominated diphenyl ether mixtures (PBDEs), organochlorine pesticides, and the elements arsenic (As), cadmium (Cd), mercury (Hg), and lead (Pb) (mean, minimum–maximum, n = 4) showing up-concentration or dilution during processing.

	Oil	Meal		Oil	Meal
	Mean	Mean		Mean	Mean
	(min–max)	(min–max)		(min–max)	(min–max)
Sum (PCDD+PCDF) (UB)	2.2	1.3	sum aldrin dieldrin (UB)	3.8	0.7
	(2.0–2.3)	(1.2–1.6)		(2.5–5.3)	(0.6–0.8)
Sum (PCDD+PCDF) (LB)	4.5	0.2	sum aldrin dieldrin (LB)	5.1	0.6
	(4.0–5.5)	(0.0–0.3)		(3.0–5.6)	(0.5–0.8)
DL-PCBs (UB)	3.9	0.7	sum chlordane (UB)	0.7	0.6
	(3.1–3.4)	(0.6–0.8)		(0.6–0.8)	(0.6–0.7)
DL-PCBs (LB)	3.9	0.4	sum chlordane (LB)	1.1	0.6
	(3.1–3.4)	(0.05–0.6)		(0.4–1.3)	(0.5–0.7)
Sum PCDD/F DL-PCBs (UB)	2.6	1.2	As	0.6	0.6
	(2.3–2.9)	(1.0–1.5)		(0.6–0.7)	(0.5–0.8)
Sum PCDD/F DL-PCBs (LB)	4.1	0.3	Cd		1.1
	(3.4–4.4)	(0.05–0.5)			(0.8–1.5)
PCB-6 UB	3.6	0.9	Hg		1.6
	(3.3–3.7)	(0.7–1.0)			(1.5–2.0)
PCB-6 LB	3.6	0.7	Fe		1.8
	(3.3–3.8)	(0.5–0.8)			(1.5–1.8)
PBDE (UB)	4.2	1.5	Se		1.0
	(3.5–4.8)	(1.1–1.9)			(0.8–1.2)
PBDE(LB)	4.7	0.2	Mn		1.8
	(4.5–5.3)	(0–0.5)			(1.8–1.9)
HCB (UB)	3.1	0.8	Zn		1.8
	(2.5–3.3)	(0.7–0.9)			(1.6–1.9)
HCB (LB)	3.1	0.8	Cu		1.5
	(2.5–3.3)	(0.7–0.9)			(1.5–1.6)
Sum DDT/E/D (UB)	2.5	0.8	Co		0.3
	(2.3–2.6)	(0.7–0.9)			(0.0–1.0)
Sum DDT/E/D (LB)	4.4	0.5	Mo		1.2
	(4.0–4.9)	(0.4–0.8)			(0.5–2.0)
Sum HCH (UB)	0.5	1.0	F		1.2
	(0.4–0.6)	(0.8–1.2)			(1.1–4.6)
Sum HCH (LB)	4.1	0.6			
	(3.0–4.7)	(0.4–0.8)			

## Data Availability

Data can be made available on request.
